# Microglia modulate proliferation, neurite generation and differentiation of human neural progenitor cells

**DOI:** 10.3389/fcell.2022.997028

**Published:** 2022-10-13

**Authors:** Julianna Lilienberg, Ágota Apáti, János M. Réthelyi, László Homolya

**Affiliations:** ^1^ Institute of Enzymology, Research Centre for Natural Sciences, Budapest, Hungary; ^2^ Doctoral School of Biology, Institute of Biology, ELTE Eötvös Loránd University, Budapest, Hungary; ^3^ Molecular Psychiatry and in vitro Disease Modelling Research Group, National Brain Research Project, Hungarian Academy of Sciences and Semmelweis University, Budapest, Hungary; ^4^ Department of Psychiatry and Psychotherapy, Semmelweis University, Budapest, Hungary

**Keywords:** human neural progenitor cell, microglia, neurite, neural regeneration, neuronal polarization, microglia polarization

## Abstract

Microglia, the primary immune cells of the brain, significantly influence the fate of neurons after neural damage. Depending on the local environment, they exhibit a wide range of phenotypes, including patrolling (naïve), proinflammatory, and anti-inflammatory characteristics, which greatly affects neurotoxicity. Despite the fact that neural progenitor cells (NPCs) and hippocampal neurons represent cell populations, which play pivotal role in neural regeneration, interaction between microglia and these cell types is poorly studied. In the present work, we investigated how microglial cells affect the proliferation and neurite outgrowth of human stem cell-derived NPCs, and how microglia stimulation with proinflammatory or anti-inflammatory agents modulates this interaction. We found that naïve microglia slightly diminish NPC proliferation and have no effect on neurite outgrowth. In contrast, proinflammatory stimulated microglia promote both proliferation and neurite generation, whereas microglia stimulated with anti-inflammatory cytokines augment neurite outgrowth leaving NPC proliferation unaffected. We also studied how microglia influence neurite development and differentiation of hippocampal dentate gyrus granule cells differentiated from NPCs. We found that proinflammatory stimulated microglia inhibit axonal development but facilitate dendrite generation in these differentiating neurons. Our results elucidate a fine-tuned modulatory effect of microglial cells on cell types crucial for neural regeneration, opening perspectives for novel regenerative therapeutic interventions.

## Introduction

The human body contains more than 200 different types of cells, many of which exhibit an asymmetric architecture to be capable of accomplishing their functions. This is best exemplified by neurons, which have a special cell polarity. They develop from neural progenitor cells, which are multipotent, self-renewing stem cells committed to the neural lineage (Breunig et al., 2011). As an early step of neural polarization, NPCs form projections, which later evolve into an axon and dendrites. This polarized architecture is prerequisite for neural communication. Besides populating various brain regions during ontogeny, NPCs also play an important role in neural regenerations after injury (Duncan and Valenzuela, 2017; Lane et al., 2017); however, this neural repair is fairly limited by different extrinsic and intrinsic factors. Inflammation for instance can adversely affect the regeneration (Ekdahl et al., 2009; Kerschensteiner et al., 2009). Depending on the severity of the injury, the local cells produce proinflammatory factors, such as cytokines, chemokines and other small molecules, which can impair neurogenesis. Several studies demonstrated that the NPCs are also present in the adult mammalian brain (Kempermann et al., 1997; Bonfanti, 2016). Neural differentiation occurs mainly in two brain regions, i.e., the subventricular zone of the lateral ventricle and the subgranular zone of the hippocampal dentate gyrus (DG) (Gage, 2000). NPCs located in the subventricular zone differentiate into interneurons and migrate into the olfactory bulb (Kaneko et al., 2017), while freshly formed neurons in the hippocampal dentate gyrus have a role in learning and in memory formation (De Miranda et al., 2017).

During the fetal development, a group of macrophage progenitors migrates into the CNS. These cells co-develop there with NPCs and gain the unique character of microglial cells (Ginhoux et al., 2010; Schulz et al., 2012). Microglia are the caretakers of the neurogenic niche, as they constantly scan the microenvironment to remove harmful substance or damages (Nimmerjahn et al., 2005). Thus, they have a pivotal role in both embryonic (Cunningham et al., 2013) and adult (Diaz-Aparicio et al., 2020) neurogenesis. They also affect synaptic refinement (Weinhard et al., 2018) and axonal guidance (Reemst et al., 2016). It has been proposed for long that microglia dwell in “resting state” under normal physiological conditions but get activated when damage occurs, acquiring either neurotoxic (M1) or neuroprotective (M2) phenotype (Prinz and Priller, 2014; Silver et al., 2015). However, our recent understanding of microglial polarization outlines a more complex scenario than the binary M1/M2 system (Hanisch, 2013; De Biase et al., 2017). Unlike “resting state” immune cells in the circulation, naïve microglia actively patrol around in the healthy brain. Damages or injuries compromising the brain homeostasis stimulate these cells, which can acquire a wide range of phenotypes depending on the incoming signals in the microenvironment.

The neuron-microglia interaction is extensively studied and well-documented [see recent reviews in (De Schepper et al., 2020; Cserep et al., 2021; Perez-Rodriguez et al., 2021)]. Despite the fact that both neural progenitors and microglia have been shown to play an essential role in neural development and regeneration (Gage, 2000; Nimmerjahn et al., 2005; Cunningham et al., 2013; Marshall et al., 2014; Diaz-Aparicio et al., 2020), only few studies explored the interaction between these cell types (Cunningham et al., 2013; Farrell et al., 2016; Choi et al., 2017; Chounchay et al., 2020). The significance of this interaction is well exemplified by the observation that microglial cells have a regulatory role in NPC distribution during adult neurogenesis (Sierra et al., 2010). In the developing brain, microglia also restrain the number of neural precursor cells, especially, microglia stimulated with lipopolysaccharide (LPS) were neurotoxic to the progenitors (Cunningham et al., 2013). Microglia activated with LPS suppresses NPCs’ viability and differentiation *in vitro* (Farrell et al., 2016). In contrast, microglia under hypoxic conditions enhance proliferation and differentiation of neural progenitors after ischemic injury (Choi et al., 2017; Chounchay et al., 2020). These observations make clear that outcome of microglia-NPC interaction greatly depends on the microenvironment, and in connection with this, the activation state of microglia. While the impact of microglia on NPCs’ proliferation/survival and differentiation was fairly studied, little is known how these immune cells influence neural cell polarity of NPCs. Chronically activated microglia have been shown to impede neurite outgrowth from these cells (Farrell et al., 2016). It is important to note that the mentioned studies are mostly based on animal models (mouse, rat, and macaque); information on the interaction between human NPCs and microglia is rather sparse. Lee et al. (2015) demonstrated that human NPCs, when implanted into the mouse brain, reduce microgliosis *via* secretion of anti-inflammatory factors and direct deactivation of proinflammatory microglia. However, how microglia act on human neural progenitor is still elusive.

In the present work, we investigated the modulatory effect of microglial cells on the polarization and differentiation of human stem cell-derived NPCs. We examined how naïve microglia influence the neurite generation and proliferation capacity of the NPCs, and how this interaction is altered when microglia are pre-treated with various pro- or anti-inflammatory agents. We also studied the impact of microglial cells on the neurite development of NPCs differentiating toward DG granule cells.

## Materials and methods

### Neural progenitor cells and their culturing

Human neural progenitor cells were previously generated from an induced pluripotent stem cell line (62F, obtained from Fred H. Gage, Salk Institute, United States) using an established directed differentiation protocol (Yu et al., 2014) slightly modified by (Lilienberg et al., 2021). Also in a previous work, the green fluorescent protein (GFP) was stably expressed in these NPCs using a Sleeping Beauty transposon-based gene delivery system (Kolacsek et al., 2014), and the cells expressing GFP at high levels were sorted out by flow cytometry, establishing the GFP-NPC line (Lilienberg et al., 2021).

For the recent study, the formerly generated neural progenitor cells lines (parental and GFP-expressing NPCs) were maintained on 6-well plates (Greiner) previously coated with poly-ornithine (pOrn, Sigma) and laminin (Lam, Thermo Fisher Scientific). For coating, plates were incubated with 2 ml/well of 10 μg/ml pOrn in phosphate-buffered saline (PBS, Thermo Fisher Scientific) for 24 h at RT; washed three times with PBS; and incubated with 2 ml/well of 5 μg/ml Lam for an additional 16 h at 4°C. 3–5×10^5^ cells were seeded into each well in NPC basal medium containing DMEM/F-12, GlutaMax™ supplemented with N2 Supplement-A, B27 (all from Thermo Fisher Scientific), bFGF (Invitrogen), Antibiotic-Antimycotic (Gibco), and Lam (1 μg/ml). The cells were cultured under these conditions until confluency, then were washed with PBS and passaged using accutase (Stem Cell Technologies).

For surface coating experiments, 96-well plates (Greiner) were covered with pOrn (10 μg/ml), poly-lysine (pLys, 5 μg/ml, Sigma), or the combination of pOrn and Lam (10 μg/ml and 5 μg/ml, respectively) as described above.

### Microglial cells and their culturing

To investigate how microglial cells affect neurite generation of human NPCs and developing human neurons, the immortalized mouse microglial cell line BV2 was used. It should be noted here that the choice of this cell line is a compromise, since application of microglia of human origin would be more appropriate. However, human primary microglia unavailable in appropriate quantity and quality; the commercially available human microglial cell lines, such as HMC3 and CHME-5, are not sufficiently characterized, and their cellular and paracrine features are questioned (Dello Russo et al., 2018). Human microglial cultures can also be differentiated from pluripotent stem cells (Muffat et al., 2016; Hasselmann and Blurton-Jones, 2020), however, the efficacy and reproducibility of these protocols are ambiguous; contaminating cells can make the results obtained with these cultures questionable. In contrast, BV2 is a stable and well-characterized cell line with a vast literature demonstrating its functional compatibility with primary microglia (Henn et al., 2009). Several studies successfully applied co-cultures of rodent and human cells interaction, demonstrating that cellular communications are evolutionarily preserved (Liu et al., 2013; Tian et al., 2021). Moreover, the modulatory effect of human neural stem cells on the microglial phenotype of murine BV2 cells has been reported (Lee et al., 2015).

BV2 cells, obtained from Ádám Dénes (Institute of Experimental Medicine, Hungary), were grown on 12 well plates (Greiner) precoated with pLys (5 μg/ml). The cells were maintained at 37°C and 5% CO_2_ in DMEM High Glucose containing 10% heat-inactivated fetal bovine serum (Thermo Fisher Scientific), 100 U/ml penicillin, and 100 mg/ml streptomycin. The cells were regularly tested for *mycoplasma* contamination.

For proinflammatory stimulation, BV2 cells were treated with human interferon gamma (IFNγ, 10 ng/ml, Peprotech) or LPS (0.1 μg/ml, Sigma) for 24 h, whereas for anti-inflammatory stimulation, the cells were subjected to human interleukin-4 (IL-4, 20 ng/ml, Peprotech) or human interleukin-13 (IL-13, 10 ng/ml, Peprotech) also for 24 h.

### Exposure of NPCs to microglia-conditioned media; NPC-BV2 co-cultures

**FIGURE 1:**
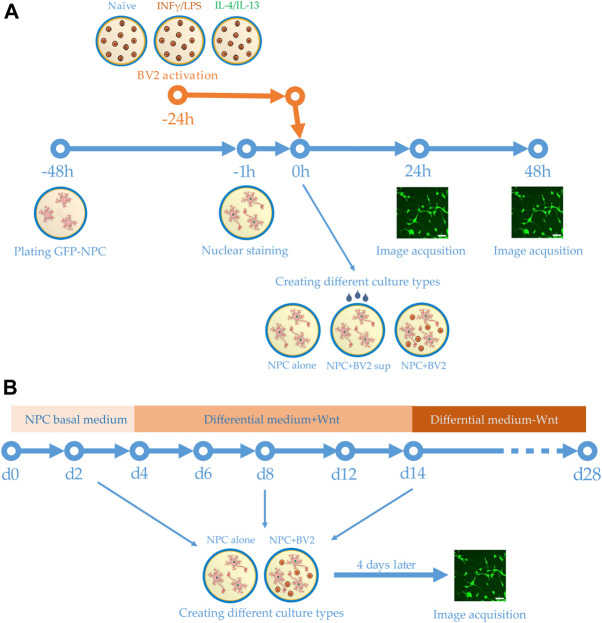
Schemes of experimental design. **(A)** Human GFP-NPCs were plated 2 days prior to the experiments. Meanwhile BV2 cells were stimulated with proinflammatory agents or anti-inflammatory cytokines. One hour before the experiments, the nuclei of GFP-NPCs were stained. GFP-NPCs were exposed to microglia-conditioned media (sup) or co-cultured with microglia. GFP-NPCs alone served as control. Images were acquired using HCS at 0, 24, and 48 h. **(B)** Schematic representation of the differentiation protocol. Human NPCs were cultured in NPC basal medium for 4 days, then further cultured for an additional 10 days in differentiation media. After 2 weeks, the Wnt was omitted from the complemented media. Co-cultures were assembled on days 2, 8, and 14; immunofluorescence staining and image acquisition were performed after 4 days of co-culturing (on days 6, 12, and 18).

GFP-NPCs were seeded onto pOrn/Lam coated 96 well plates at 3-5x10^5^ cells per well density in 150 μl of DMEM/F-12, GlutaMax™ media supplemented with N2 Supplement-A, B27, bFGF, Antibiotic-Antimycotic and Lam. The cells were allowed to expand for 2 days at 37°C and 5% CO_2_. Meanwhile mouse BV2 cells, grown on 24 well plates in 500 µl medium, were stimulated as described above. One hour before the experiments, the nuclei of GFP-NPCs were stained with 25 nM DyeCycle Violet (DCV, Thermo Fisher Scientific). At time zero GFP-NPCs were subjected to supernatants collected from the BV2 cells 1 day after stimulation (50 µl supernatant to 150 µl medium). For co-culture experiments, BV2 cells were collected 24 h after stimulation, and seeded in 1:8 ratio (BV2:NPC) onto GFP-NPCs previously stained with DCV. Images were acquired using a high content screening microscope (HCS) at time 0, 24, and 48 h (Figure 1A).

### High content screening and analysis

GFP-NPC cultures, in either mono- or co-culture configuration, were investigated using an ImageXpress Micro XLS high content screening device (Molecular Devices). Six fields of view (approximately half of the total well surface area) were imaged using a DAPI filter cube (ex. 377/50 nm, em. 447/60 nm), a FITC filter cube (ex. 482/35 nm, em. 536/40 nm) with a 10× Nikon objective (Plan Fluor, NA = 0.3). Total outgrowth and cell number was quantified from the green and blue (GFP and DCV) fluorescence images, respectively (Lilienberg et al., 2021). Average neurite length per cell was calculated by dividing the total neurite length by the cell number. All data were normalized to the initial values (time 0) of the control (untreated NPCs or NPCs with no co-culturing). At least three biological parallel experiments (with 3 technical parallels each) were evaluated. For statistical analyses, two-way ANOVA with replications was performed considering ‘time’ and ‘treatments’ as independent variables. To identify pairwise significant differences, Tukey’s HSD post hoc test was used. Differences were considered significant when *p* < 0.05.

To evaluate neurite development in differentiating DG granule cells, high content analysis was performed with confocal immunofluorescence images (see immunostaining procedure below), and the average neurite lengths per cell were determined as described above. At least 1000 cells were evaluated for each condition. For statistical analyses, the non-parametric Mann-Whitney U test was used. Differences were considered significant when *p* < 0.05.

### Assessment of para-nitroblebbistatin-induced neurite outgrowth

BV2 cells were stimulated with proinflammatory agents (a combination of 10 ng/ml IFNγ and 0.1 μg/ml LPS), or anti-inflammatory cytokines (a combination of 20 ng/ml IL-4 and 10 ng/ml IL-13), or left unstimulated. Co-cultures of GFP-NPCs and BV2 cells were subjected to 10 µM para-nitroblebbistatin (obtained from András Málnási-Csizmadia, Eötvös Loránd University, Hungary). Images were acquired every 15 min for 4 h using a high content screening microscope. The rate of neurite outgrowth was determined as described previously (Lilienberg et al., 2021).

### Neural differentiation

NPCs without GFP were differentiated toward hippocampal DG granule cells using a previously published protocol (Yu et al., 2014; Vofely et al., 2018). Briefly, 1.5 × 10^4^ NPCs were seeded onto pOrn/Lam coated 8 well chambers (Ibidi) and cultured in the NPC basal medium. On day 4, the NPC basal medium was supplemented with ascorbic acid (200 ng/ml, Sigma), BDNF (20 ng/ml, Peprotech), cAMP (500 μg/ml, Sigma), Lam (1 μg/ml), and Wnt3a (20 ng/ml, R&D Systems). The medium was changed every other day. From day 14, the Wnt3a was omitted from the culturing medium (Figure 1B). Differentiation was concluded after 4 weeks.

### Immunofluorescence

Human NPCs seeded onto various surfaces and hippocampal DG granule cell cultures differentiated from NPCs were fixed with 4% PFA (Thermo Fisher Scientific) in DPBS for 15 min at RT. After several washing steps with DPBS, the cells were blocked for 60 min at RT with DPBS containing 2 mg/ml bovine serum albumin, 1% fish gelatin 5% goat serum and 0,1% Triton X-100 (blocking buffer, all from Sigma). Cells were then incubated overnight at 4°C with rabbit anti-βIII-tubulin (1:500, Abcam), mouse anti-tau1 (1:500, Merck), mouse anti-MAP2 (1:500, Merck), or rabbit anti-PROX1 (1:500, Abcam) primary antibodies. Samples were washed with DPBS then incubated with one or two of the following reagents (all from Thermo Fisher Scientific and diluted in blocking buffer): Alexa Fluor-488 conjugated Phalloidin (1:500); Alexa Fluor −547 or Alexa Fluor-647 conjugated anti-rabbit (1:250), Alexa Fluor-488 or Alexa Fluor-647 conjugated anti-mouse (1:250) secondary antibodies as indicated. The samples were washed with DPBS and counterstained with 1 μg/ml DAPI for 1 min at RT. As controls for antibody specificity, samples were prepared as described above with the exception of incubation with the primary antibodies. The cultures were imaged by a Zeiss LSM 710 confocal laser scanning microscope using a Plan-Apochromat 20× (NA = 0.8) and 40× (NA = 1.4) objectives.

## Results

### Neurite generation of neural progenitor cells on different extracellular matrix components

The neural progenitor cell lines used in this study have previously been generated and characterized in detail (Yu et al., 2014; Vofely et al., 2018; Lilienberg et al., 2021). These cells express no pluripotency markers, such as Nanog and Oct4; but the typical neural progenitor markers, such as Nestin and SOX2; as well as the neuronal markers, such as NeuroD1, FOXG1, and PAX6 (Yu et al., 2014; Vofely et al., 2018). These NPCs are primed to differentiate preferentially into hippocampal DG granule cells, therefore denoted as “hippocampal NPCs” (Yu et al., 2014; Vofely et al., 2018). They can be maintained with unaltered phenotype up to 15 passages (Vofely et al., 2018) and stably transfected with various transgenes, such as GFP and the fluorescent calcium indicator GCaMP6b (Vofely et al., 2018; Lilienberg et al., 2021).

Since NPCs are anchorage-dependent cells requiring surface attachment for proper development (Merten, 2015), first we investigated the impact of various extracellular matrix components (ECM) on neurite generation. ECMs not only provide structural stability but also mediate various stimuli controlling diverse cellular functions, such as cell differentiation or response to injury. GFP-expressing NPCs were seeded onto 96 well culture plates previously coated with pLys, pOrn, or the combination of pOrn/Lam, and the neurite outgrowth was studied (Supplementary Figure S1). As controls, cells grown on culture plates with no extra treatment were used. We found that all coating types supported cell attachment and prompted NPCs to form small clusters. Immunostaining for βIII-tubulin and F-actin revealed that actin accumulated only at focal adhesions in NPCs grown on non-coated surface, whereas a number of projections developed, when cells were cultured on various ECMs. In terms of neurite outgrowth, pOrn/Lam coated surface seemed to be the best (Supplementary Figures S1A,B). As regards the kinetics, shortly after seeding, NPCs regain normal morphology, having relatively short processes, the length of which hardly changes for the following 24-36 h but starts elongate 48 h after seeding. Quantitative analysis revealed that pOrn/Lam coating supports NPCs to generate significantly longer neurites as compared to cells plated on non-treated or pLys-coated surface (Supplementary Figure S1C); therefore, pOrn/Lam coating was used in the subsequent experiments. It is worth mentioning that GFP expression caused no alteration in the cell morphology or the proliferative capacity of NPCs (Lilienberg et al., 2021).

### Effect of naïve microglia on human neural progenitor cells

**FIGURE 2:**
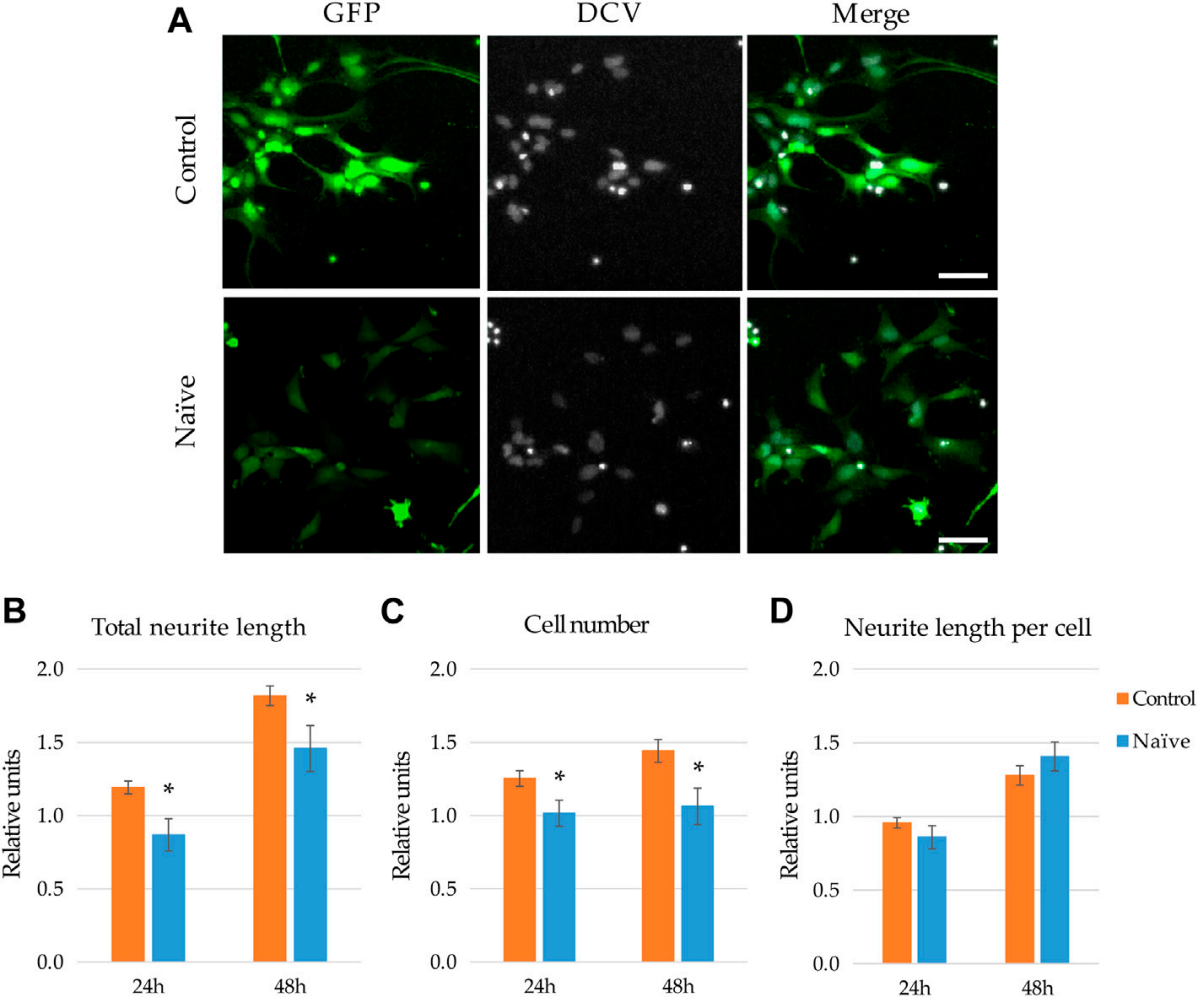
Effect of naïve microglia on the neurite outgrowth and proliferation of NPCs. **(A)** Representative fluorescence images depict GFP-expressing NPCs (upper panels) and counterparts exposed to the supernatant of naïve BV2 cells for 48 h (lower panels). GFP fluorescence (green) and counterstaining with the nuclear dye DCV (grey) are shown. Scale bars: 25 µm. For quantitation, the total neurite lengths **(B)**, the cell number **(C)**, and the neurite length per cell **(D)** were determined at 24 and 48 h. The relative units represent the values normalized to time 0 points of untreated samples. Data are presented as mean ± SEM (n = 8). For statistical analysis, two-way ANOVA followed by Tukey’s HSD post hoc test was performed. Asterisks indicate significant differences as compared to untreated cells (p < 0.05).

To investigate how naïve microglia influences neurite generation of NPCs, GFP-expressing human neural progenitor cells were subjected to the supernatant of BV2 cells. Total neurite outgrowth at different time points was determined on the basis of green fluorescence and morphological parameters, whereas the corresponding cell numbers of GFP-NPCs were quantified based on the nuclear staining (Figure 2A). We found that untreated GFP-NPCs 48 h after plating gradually develop protrusions at a rate of 7.09 ± 2.03 mm/cell/48 h (∼80% increase within the following 48 h), which is consistent with our previous findings on neurite growth rate of unstimulated GFP-NPCs grown on pOrn/Lam coated surface (Lilienberg et al., 2021). NPCs under the applied conditions exhibit a moderate proliferation rate (cell number increased by ∼20% per day, the equivalent of a doubling time of 4 days). Treatment with the supernatant of naïve microglia slightly decelerated total neurite development (Figure 2B). In parallel, this treatment modestly inhibited NPCs’ proliferation (Figure 2C), although 2-3% increase in cell number was still observed, implying that the naïve microglia supernatant was not toxic to NPCs. Since both the total neurite growth and cell proliferation was reduced to a similar extent, the neurite length per cell remained unchanged (Figure 2D). We also investigated this interaction in co-cultures to explore weather cell-cell contact adds an extra effect or acts differently. Various NPC-microglia ratios were initially studied (1:16, 1:8, 1:4, 1:2), which resulted in similar results, although 1:4 and 1:2 were proven to be technically challenging due to the high proliferation rate of BV2 cells. Therefore, 1:8 cell ratio was used in the subsequent co-culturing experiments. Nevertheless, similar results were obtained with co-cultures to that seen with the BV2 supernatants: naïve microglia had no major effect on the neurite development (Supplementary Figures S2A,C), and were not toxic to NPCs, since co-culturing did not affect NPCs’ proliferation (Supplementary Figure S2B).

### Effect of activated microglia on the neurite generation of neural progenitor cells

Disruption of brain homeostasis results in a quick change in the microglial phenotype from a naïve state to an activated condition. As a response to external signals, microglia alter their morphology, surface receptor repertoire, as well as production and secretion of various paracrine molecules. These phenotypic changes greatly depend on the incoming stimuli. To examine the effect of reactive microglia cells on NPCs, BV2 cells were treated with proinflammatory agents, such as IFNγ and LPS, or alternatively with anti-inflammatory agents, such as IL-4 and IL-13. Upon stimulation BV2 cells underwent marked morphological changes: their flat, spindle-like cell shape became more round in response to LPS, whereas turned into elongated ones with more processes when stimulated with IL-4 (Supplementary Figure S3).

**FIGURE 3:**
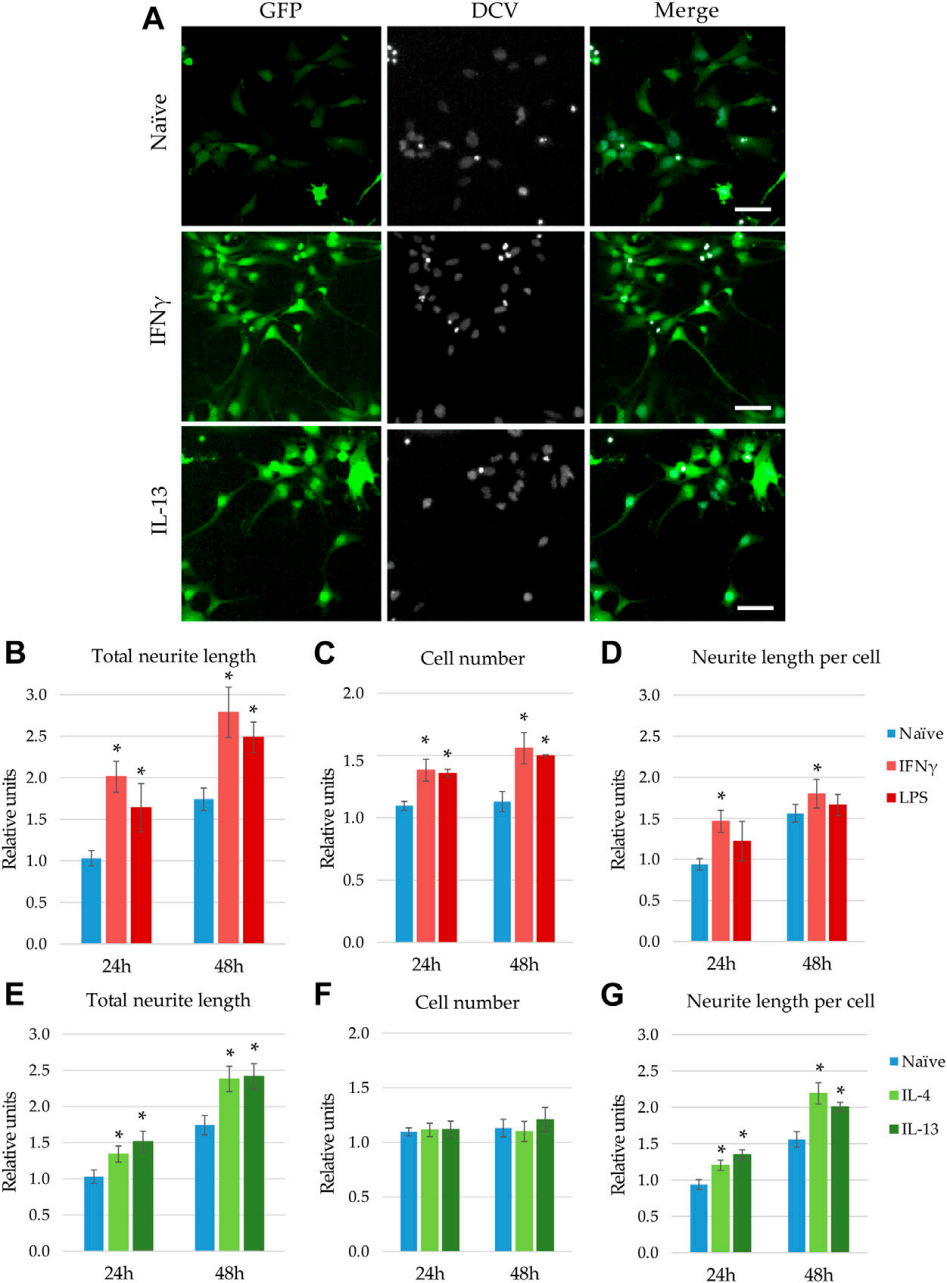
Modulatory effect of microglia stimulation on microglia-NPC interaction. GFP-NPCs were subjected to supernatants from BV2 cells previously stimulated with proinflammatory agents (IFNγ or LPS), or anti-inflammatory cytokines (IL-4 or IL-13). As controls, GFP-NPCs treated with supernatants from naïve BV2 cells were used. **(A)** Representative fluorescence images depict GFP fluorescence (green) and DCV counterstaining (grey) in GFP-NPCs exposed to naïve, IFNγ-, or IL-13-pretreated BV2 supernatants for 48 h. Scale bars: 25 µm. The total neurite lengths **(B,E)**, the cell numbers **(C,F)**, and the neurite lengths per cell **(D,G)** were determined at the indicated time points. The relative units represent the values normalized to time 0 points of untreated NPCs. Data are presented as mean ± SEM (*n* = 4–7). For statistical analysis, two-way ANOVA followed by Tukey’s HSD post hoc test was performed. Asterisks indicate significant differences as compared to treatment with naïve BV2 supernatants (*p* < 0.05).

When GFP-NPCs were exposed to supernatants from BV2 previously stimulated with IL-13, augmented neurite generation was observed (Figure 3A). Surprisingly, supernatants from IFNγ -treated microglia also accelerated GFP-NPCs’ neurite development. Similar morphological changes were observed in response to treatment with LPS and IL-4 (data not shown). To quantitate these observations, high content screening and analysis was performed. In concert with qualitative observations we found accelerated neurite generation in GFP-NPCs exposed to supernatants from microglia stimulated with either IFNγ or LPS (Figure 3B). In GFP-NPCs treated with naïve BV2 supernatant, total neurite length increased 1.75-fold within 48 h, whereas conditioned media from IFNγ- or LPS-stimulated BV2 cells augmented neurite growth 2.7- and 2.4-fold, respectively. In parallel, the cell numbers were determined, and the neurite lengths per cell were calculated. Surprisingly, proinflammatory microglia was not toxic to NPCs. As compared to naïve microglia-conditioned medium, supernatants from either IFNγ- or LPS-treated BV2 cells slightly stimulated GFP-NPC proliferation (Figure 3C), as well as augmented neurite outgrowth at the single cell level (Figure 3D). To examine whether the observed accelerated neurite generation and proliferation were due to the direct effect of the proinflammatory agents or caused by other substances in the BV2-conditioned media, GFP-NPCs were directly subjected to IFNγ or LPS. The former had no effect; the latter had a slight inhibitory effect on neurite generation of GFP-NPCs, whereas neither IFNγ nor LPS affected GFP-NPC proliferation (Supplementary Figure S4).

Next, we investigated how microglia stimulated with anti-inflammatory cytokines influence neurite generation (Figures 3E,G). Consistent with the qualitative observation shown in Figure 3A, high content screening and analysis demonstrated that supernatants from BV2 cells pretreated with IL-4 or IL-13 significantly stimulate neurite generation in GFP-NPC cultures (Figure 3E). Contrary to conditioned media from IFNγ- or LPS-activated microglia, these supernatants did not affect GFP-NPC proliferation (Figure 3F); thus, increase in total neurite growth was due to augmented neurite generation at the single cell level (Figure 3G). It is important to note that IL-4 and IL-13 have no direct effect on neurite generation and proliferation of GFP-NPCs (Supplementary Figure S4).

To explore whether cell-cell contact can further modulate the interaction between neural progenitor cells and microglia, we co-cultured GFP-NPCs with BV2 cells stimulated either with pro- or anti-inflammatory agents. Similar to supernatant treatments, co-culturing with microglia pre-activated with IFNγ or LPS markedly stimulated neurite generation and slightly increased proliferation of GFP-NPCs (Supplementary Figures S5A–C). BV2 cells pretreated with IL-4 or IL-13 also stimulated neurite growth leaving GFP-NPC proliferation unaffected (Supplementary Figures S5D–F). Since the effect of microglia supernatants on GFP-NPCs were qualitatively comparable with that seen in the co-cultures, in further experiments we used only the former experimental configuration.

Hitherto, we investigated how microglia influence normal development of neurites of human neural progenitor cells. In a previous study, we demonstrated pharmacological modulation of neurite outgrowth in GFP-NPCs (Lilienberg et al., 2021) The non-muscle myosin II inhibitor, blebbistatin and its derivatives, such as para-nitroblebbistatin (NBS) and para-aminoblebbistatin, elicited extensive generation of neurites. Thus, we have found noteworthy to explore whether microglia modulate the effect of blebbistatin on GFP-NPCs’ neurite outgrowth. These examinations required slightly different experimental approach, since blebbistatin-induced neurite generation was much faster than normal neurite development. Images were acquired every 15 min for 4 h, and the initial rates of neurite outgrowth were determined from the kinetic curves (Lilienberg et al., 2021). Surprisingly, co-culturing of GFP-NPCs with naïve microglia further enhanced NBS-induced neurite outgrowth (Supplementary Figure S6). Similar effect was obtained, when GFP-NPCs were co-cultured with IL-4/IL13-pretreated BV2 cells. However, pre-activation of microglia with IFNγ and LPS prevented this augmentation.

### Impact of microglia on neurite generation of differentiating neuron

In the human adult brain, neural progenitor cells reside primarily at two sites, i.e., the subventricular zone and the hippocampal dentate gyrus (Martinez-Cerdeno and Noctor, 2018). When damage occurs, NPCs migrate from these reservoirs, start to proliferate and differentiate to maintain the homeostasis of the CNS. During this process, NPCs may encounter microglial cells, especially at the site of injury. To understand the nature of this pathophysiological interaction better, in the next set of experiments, we investigated whether microglia influence neurite development of neural cells differentiating from NPCs (Figure 1B). For this set of experiments, we applied the original NPC line, which has no GFP expression, for its fluorescence may interfere with multicolor immunofluorescence staining. As mentioned earlier, NPCs used in this study can be differentiated into hippocampal DG granule cells. In the fully differentiated cultures, most of the cells (85%–100%) are glutamatergic, and a major part (55%–70%) expresses PROX1, a hallmark of DG granule cells (Yu et al., 2014; Vofely et al., 2018). These differentiated cells also express several other typical markers including NeuroD1, DCX, TBR1 and CALB1 (Yu et al., 2014; Vofely et al., 2018).

**FIGURE 4:**
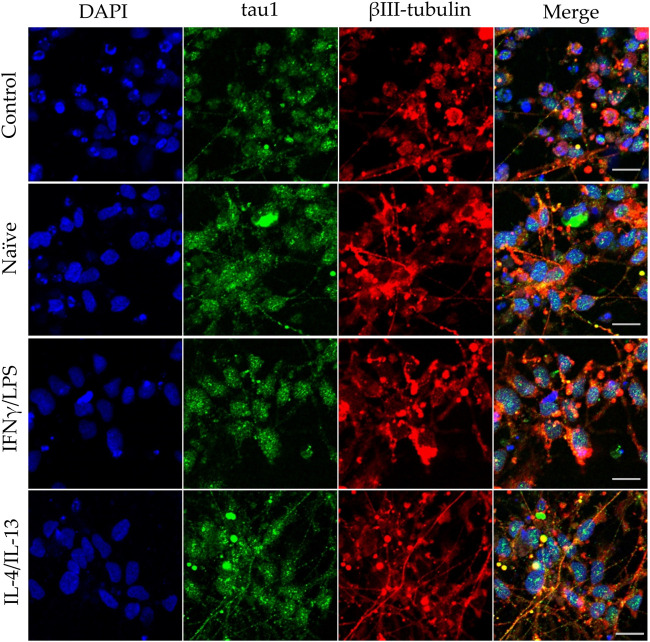
Impact of naïve and stimulated microglia on the axon development of differentiating neurons. NPCs were differentiated toward the hippocampal DG granule cells for 18 days. The differentiating neuronal cells were co-cultured with BV2 cells for 4 days (from day 14 to day 18). Microglial cells remained untreated (naïve) or were previously stimulated with an IFNγ/LPS or an IL-4/IL-13 cocktail as indicated. Confocal images depict day 18, neuronal cultures immunostained for the axonal marker tau1 (green) and the neuronal marker βIII-tubulin (red). The samples were counterstained with DAPI (blue). Scale bars: 25 µm.

**FIGURE 5:**
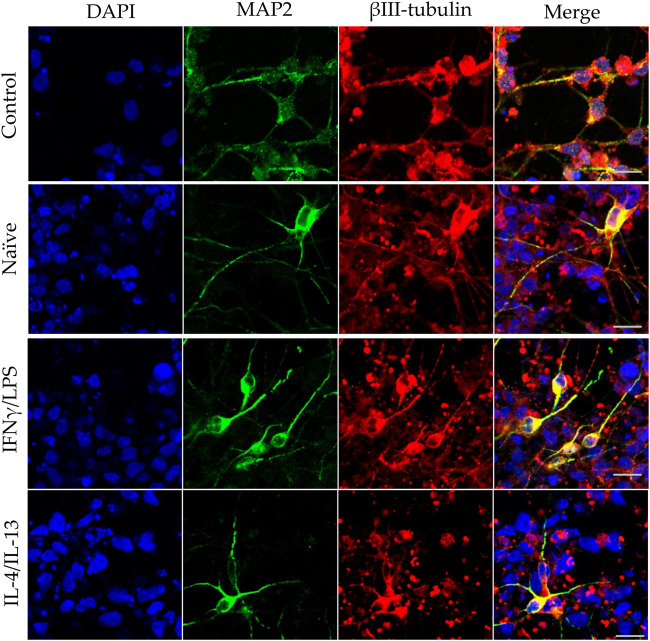
Effect of naïve and stimulated microglia on the dendrite development in differentiating neurons. NPCs were differentiated toward the hippocampal DG granule cells for 18 days. The differentiating neuronal cells were co-cultured with BV2 cells for 4 days (from day 14 to day 18). Microglial cells remained untreated (naïve) or were previously stimulated with an IFNγ/LPS or an IL-4/IL-13 cocktail as indicated. Confocal images depict day 18 neuronal cultures immunostained for the dendritic marker MAP2 (green) and the neuronal marker βIII-tubulin (red). The samples were counterstained with DAPI (blue). Scale bars: 25 µm.

**FIGURE 6:**
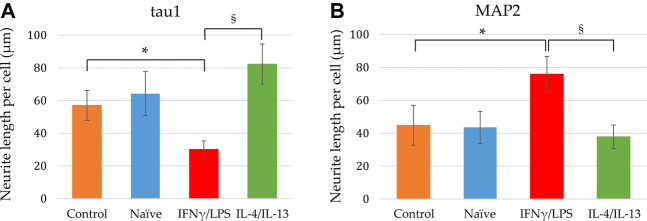
Quantitative analysis of axonal and dendritic development of differentiating neuronal cells exposed to naïve and stimulated microglia. Using confocal images similar to that shown in Figures 4, 5, the tau1-positive **(A)** as well as MAP2-positive **(B)** neurite lengths per cell were calculated from the corresponding total neurite lengths and the cell numbers. Data are presented as mean ± SEM calculated from two biological parallels (each conditions include 1000–2500 cells). For statistical analysis, Mann-Whitney U test was performed. Asterisks indicate significant differences as compared to control (NPCs with no co-culturing), whereas section signs mark significant differences between neuronal cells co-cultured with pro- and anti-inflammatory stimulated microglia (*p* < 0.05).

In our experiments, we differentiated these “hippocampal NPCs” toward hippocampal DG granule cells, and co-cultured with naïve or stimulated BV2 cells at various phases of differentiation (on days 2, 8, and 14) for 4 days. To demonstrate the lineage commitment, some of the cultures (not exposed to microglia) were further differentiated and immunostained for PROX1. After 4 weeks of differentiation, the majority of the cells were positive for PROX1 staining (Supplementary Figure S7). When differentiating DG granule cells were co-cultured with microglia, the cell survival was not affected (data not shown). The complexity of developed neurite network did not allow us to assess the total neurite length by high content screening, as performed in the previous experiments with GFP-NPCs. Thus, we rather imaged co-cultures of differentiating neural cells by confocal microscopy. Neural cells were immunostained for the neuron-specific marker βIII-tubulin, as well as two types of microtubule-associated proteins: tau1, which is highly enriched in axons, and MAP2 present in dendrites (Paglini et al., 2000). On day 6 of differentiation, tau1 expression was detected mainly in the nucleus and to some extent, in the cytoplasm of differentiating neural cells (Supplementary Figure S8). It is worth noting that at this phase, neural cells co-cultured with IFNγ/LPS stimulated microglia exhibited round cell morphology. After additional 6 days, the cultures underwent marked morphological changes, but tau1 still localized predominantly to the nucleus and the cytoplasm (Supplementary Figure S10). By day 18, tau1 was observed not only in the cytoplasm but also in certain neurites, presumably developing axons (Figure 4). All culture configurations exhibited tau1 accumulation in the processes although to various extent. Quantitative analysis of confocal images demonstrated that co-culturing with naïve microglia did not affect axon generation in differentiating neural cells, however, proinflammatory stimulated microglial cells significantly impaired the process, whereas co-culturing with IL-4/IL-13 stimulated microglia seemed to be slightly stimulatory (Figure 6A). Similarly, the dendritic marker MAP2 on days 6 and 12 exhibited intracellular distribution in differentiating neural cells (Supplementary Figure S9, 11). By day 18, MAP2 was profoundly localized to processes (Figure 5). Quantitation demonstrated that co-culturing with IFNγ/LPS stimulated microglia augmented dendrite generation in differentiating neurons as compared to cultures not exposed to BV2 cells (Figure 6B). Co-culturing with naïve or IL-4/IL-13 stimulated microglia had no effect on the dendrite development of differentiating neural cells. Taken together, these results suggest proinflammatory microglia alter the differentiation of neurites of developing neurons, and modify the axonal-dendritic balance.

## Discussion

Despite the fact that neural progenitor cells represent a cell type that has an essential role in neural development and regeneration, most studies published thus far are based on non-human mammalian, mainly rodent models (Amato et al., 2011; Bi et al., 2014; Abbott and Nigussie, 2020; Larson et al., 2020). On the other hand, studies using NPCs of human origin mostly focused on their regenerative capacity for potential medical applications (Nagoshi et al., 2019; Tsuji et al., 2019; Kajikawa et al., 2020). In contrast, we investigated human NPCs from a developmental aspect, exploring the intrinsic and external factors that influence the initial events of neural polarization: neurite generation and differentiation. Previously, we demonstrated that non-muscle myosin II plays a pivotal role in neurite outgrowth of human NPCs (Lilienberg et al., 2021). We also showed that its pharmacological inhibition is capable of overriding the inhibitory effect of detrimental extracellular matrix components, which are characteristically present at the site of injuries (Lilienberg et al., 2021).

In the present work, we explored the modulatory effect of microglia on neurite generation of NPCs, and found that unlike naïve microglia, proinflammatory stimulated microglia augmented both NPC proliferation and neurite generation. This finding is in contrast with previous observations made with mature neurons isolated after spinal cord injury (SCI), where proinflammatory microglia facilitated axonal retraction (Horn et al., 2008; Kitayama et al., 2011). Interestingly, it has been reported that dual activation with IFNγ and LPS was required for the effect; stimulation with IFNγ alone was not sufficient (Horn et al., 2008). In our case, IFNγ-treated microglia were as effective as the LPS-treated ones. On the other hand, in concert with our observation, proinflammatory stimulated microglia have been reported to promote axon growth in cortical neurons isolated from newborn mouse pups (Kigerl et al., 2009); as well as IFNγ-treated microglia have been shown to stimulate neuronal differentiation (Butovsky et al., 2006). Our finding that anti-inflammatory microglia stimulated neurite generation in human NPCs is also in agreement with previous observations made in other models. IL-4 treated microglia have been reported to stimulate axonal growth (Kigerl et al., 2009), neurogenesis, as well as neuronal and oligodendrocyte differentiation (Butovsky et al., 2006; Choi et al., 2017; Yuan et al., 2017). IL-4 treatment of co-cultures of motoneuron and microglia also protects neuronal cells from neurite shortening induced by toxic conditions (Zhao et al., 2006). It is also important to note that the applied agents (IFNγ, LPS, IL-4, IL-13) had no marked direct effect on the proliferation and neurite generation of NPCs (Supplementary Figure S4), which is in concert with previous studies demonstrating lack of impact of IFNγ, LPS, and IL-4 on mouse NPCs and neurons (Butovsky et al., 2006; Kigerl et al., 2009). Conclusively, microglia may differentially affect neural cells depending on the cell type, their maturity, and possibly their stress related condition, e.g., preceding exposure to injury or ischemic stroke. Nevertheless, our results further support the previously proposed notion that the “neurotoxic” term commonly used for proinflammatory stimulated microglia should be revised.

Our results demonstrated the modulatory effect of microglia also on pharmacologically elicited neurite outgrowth in NPCs. Surprisingly, naïve and anti-inflammatory microglia further accelerated NBS-induced neurite growth, whereas proinflammatory microglia had no effect. The most plausible explanation for the observed difference between effects of microglia on normal and drug-induced neurite outgrowth is that the underlying mechanisms of neurite generation elicited by non-muscle myosin II inhibition greatly differ from the physiological or pathophysiological situations. Nevertheless, our results draw attention to the potential synergistic effect of microglia on pharmacologically induced neurite outgrowth.

There are conflicting results on whether cell-cell contact is necessary for the impact of microglia on the neurite lengths of a neural cell. Horn et al. (2008) reported that axonal retraction in SCI neurons caused by LPS-treated microglia does not require cell-cell contact, whereas Kitayama et al. (2011) showed that these impact occurs only when the two cell types are closely co-cultured. On the other hand, stimulated microglia promoted axonal growth in cortical neurons without cell-cell contact (Kigerl et al., 2009). To elucidate whether interaction between microglia and NPCs requires cell-cell contact, we applied two major configurations in our experiments: co-culturing and exposure to supernatants, but no major difference between the two arrangements was observed, implying that direct cell-cell contact has no significant role in microglia-NPC interaction—at least not with regards to neurite outgrowth.

Microglia, like other macrophage-like cells, are known to secrete numerous substances as main line of cell-to-cell communication. Our results imply that microglia-NPC interaction is mediated by materials secreted into the media, although it is also possible that the signal from microglia to NPCs is transmitted *via* extracellular vesicles or exosomes, which is an alternative way of cellular communication of microglial cells (Thompson et al., 2016). The immortalized murine microglial cell line BV-2 has been verified as a suitable substitute for primary microglia in terms of secreted substances (Henn et al., 2009). Unstimulated BV2 cells secrete TNFα, IL-6, and IL-12p70, but their IL-1β and IL-10 production is moderate as compared to that of primary microglia (Schmidt et al., 2021). Interestingly, unstimulated BV2 cells also secrete IGF1, which is characteristic to neonatal microglial cells (Schmidt et al., 2021). Identification of signaling molecules responsible for stimulus of NPCs’ neurite growth is rather challenging, since close to 5000 secreted proteins are associated with the proinflammatory activation of BV2 cell with LPS or IFNγ (Woo et al., 2017). The major characteristic features of proinflammatory microglia include increased level of TNFα, IL-1β, IL-6, CCR2, and CD86; elevated expression of nitric oxide synthase (iNOS) and consequently increased nitric oxide production (Henn et al., 2009; Michelucci et al., 2009; Orihuela et al., 2016; Yuan et al., 2017; Jiang et al., 2022). In contrast, anti-inflammatory microglia are characterized by increased level of Arg1, IL-4, IL-10, YM-1, CD163, and CD206 (Michelucci et al., 2009; Orihuela et al., 2016; Yuan et al., 2017; Jiang et al., 2022). Interestingly, in IFNγ stimulated microglia, after rapid decline of proinflammatory factors, the anti-inflammatory markers Arg1 and IL-10 elevate 24 and 48 h after ceasing a 24 h long stimulation (Jiang et al., 2022).

**FIGURE 7:**
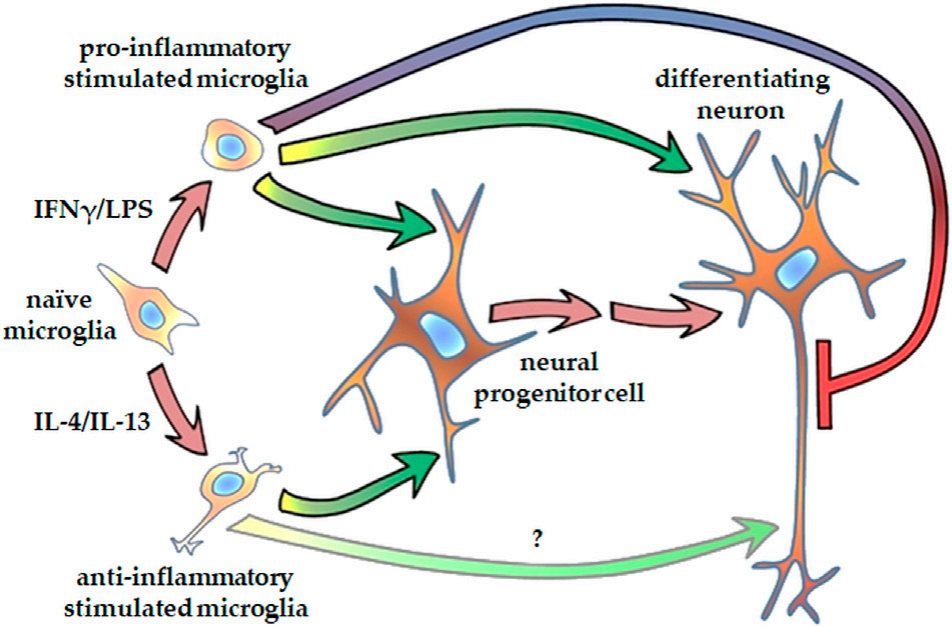
Schematic representation of how microglia affect neurite generation of human NPCs and differentiating neurons. Microglia stimulated either with proinflammatory agents, such as IFNγ and LPS, or with anti-inflammatory cytokines, such as IL-4 and Il-13, promote neurite outgrowth in human neural progenitor cells. Proinflammatory activated microglia augment dendritic development, but inhibits axon generation in differentiating hippocampal DG granule cells. Microglia stimulated with anti-inflammatory cytokines supposedly support axon generation in granule cells.

Our observation that proinflammatory stimulated microglia promote NPCs proliferation and neurite generation is rather unexpected, since reactive microglia and the proinflammatory cytokines secreted by these cells are generally considered detrimental to the microenvironment. However, TNFα, IL-1β, and IL-6 have been shown to improve the neuronal survival of cortical neurons challenged with NMDA (Carlson et al., 1999). TNFα and IL-1β also increase neurite number of enteric neurons in a dose dependent manner (Gougeon et al., 2013). Results on the effect of proinflammatory cytokines on neurite outgrowth are, however, conflicting. While IL-1β suppressed axonal development in the cortex of neonatal septic rats (Han et al., 2017), it stimulated neurite outgrowth in peripheral neurons, i.e., dorsal root ganglia (Golz et al., 2006; Temporin et al., 2008). IL-6 has also been demonstrated to augment neurite outgrowth generation in peripheral neurons (Schafer et al., 1999; Golz et al., 2006), but the presence of IL-6 receptor was required for the stimulatory effect (Schafer et al., 1999). To identify the substances that are responsible for augmentation of NPCs neurite generation, further studies are needed, which could potentially elucidate the underlying mechanism. It is noteworthy that an extra challenge for these investigations is that combined administration of these substances may elicit different (sometimes reversed) response in neurite outgrowth from that observed with single factor applications (Golz et al., 2006). After the initial step of neural polarization, when NPCs protrude relatively short, structurally comparable processes, neurites elongate and develop to axons and dendrites in later phases of differentiation. Our results demonstrated that proinflammatory stimulated microglia modulate neurite development also at this stage. When NPCs were differentiated toward hippocampal DG granule cells, elongation of tau1-positive neurites was diminished, whereas growth of MAP2-positive neurites was stimulated by proinflammatory microglia. In contrast, anti-inflammatory microglia showed a tendency to stimulate tau1-positive neurite development. Although tau1 and MAP2 are commonly considered as axonal and dendritic markers, respectively, it should be noted that at this stage of differentiation, developing neurites are structurally and functionally immature. This is supported by the observation that on day 18 of differentiation, the total lengths of tau1-positive and MAP2-positive neurites are comparable (Figure 6), which does not apply to mature neurons. Nevertheless, our finding is in good agreement with a previous study demonstrating that proinflammatory microglia result in generation of short and branching neurites in cortical neurons of newborn mice, whereas IL-4 treated microglia lead to long, uni- or bipolar neurites (Kigerl et al., 2009). Taken together, our results demonstrate that both pro- ad anti-inflammatory microglia have stimulatory effect on NPC’s neurite generation, whereas they have distinct impact on the neurite differentiation in later phase of neural polarization (Figure 7).

In conclusion, our results demonstrate the complexity of the effect of microglia on the neurite generation and differentiation human NPCs and their progenies, which interaction depends not only on the activation state of microglia but also the maturity of neural cells and the way of neurite outgrowth initiation. However, further analyses are necessary to elucidate the details of the underlying mechanisms. NPCs represent a promising treatment option for various neurological pathologies including ischemic stroke and neurodegenerative disorders. Functional integration of the transplanted cells is a key issue, which depends on not only the intrinsic program of the implanted cell but also the recipient microenvironment. Better understating of these basic cell biological events at the neural progenitor cell level could eventually contribute to development of therapeutic strategies exploiting the regenerative potential of human NPCs.

## Data Availability

The raw data supporting the conclusions of this article will be made available by the authors, without undue reservation.

## Abbreviations

DCV, DyeCycle Violet; DG, dentate gyrus; ECM, extracellular matrix component; GFP, green fluorescent protein; HCS, high content screening microscope; IFNγ, interferon gamma; IL, interleukin; iNOS, nitric oxide synthase; Lam, laminin; LPS, lipopolysaccharide; NBS, para-nitroblebbistatin; NPC, neural progenitor cell; PBS, phosphate-buffered saline; pLys, poly-lysine; pOrn, poly-ornithine; SCI, spinal cord injury.
