# Supplementary material for: Microglia modulate proliferation, neurite generation and differentiation of human neural progenitor cells
Source: Front Cell Dev Biol. 2022 Oct 13;10:997028. doi: 10.3389/fcell.2022.997028 (PMC9606406; doi:10.3389/fcell.2022.997028)
Supplement: Supplementary file 1 [file DataSheet1.pdf]

Supplementary Material

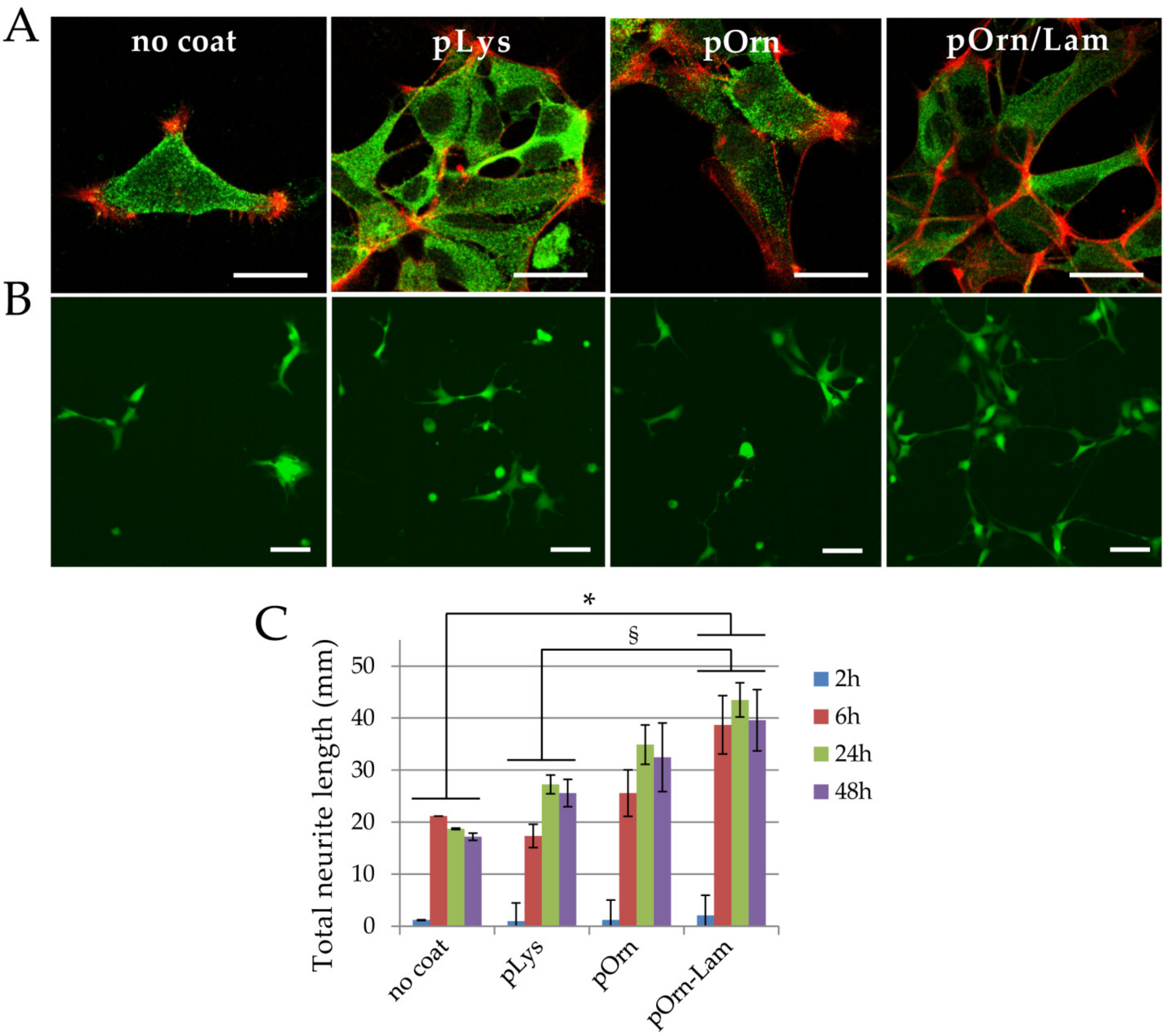

Supplementary Figure 1
Neurite outgrowth in human NPCs on various extracellular matrices

(A) Confocal fluorescence images depict NPCs seeded on non-treated 8-well chambers or surfaces coated with pLys, pOrn, or the combination of poly-ornithine and laminin (pOrn/Lam). The cells were fixed and immunostained for βIII-tubulin (green) and F-actin (red). Scale bars: 25 µm. (B) Representative images of human GFP-NPCs cultured for 24 h on 96-well plates coated with different extracellular matrix components including pLys, pOrn, and pOrn/Lam. As a control, GFP-NPCs grown on a surface with no extra treatment were used. Scale bars: 25 µm. (C) The total neurite length of GFP-NPCs grown on different ECMs was quantified 2, 6, 24, and 48 h after seeding. Data are presented as mean ± SEM (n=3). For statistical analysis, two-way ANOVA followed by Tukey's HSD post hoc test was performed. Asterisk and section mark denote significant differences as compared to uncoated and pLys-coated surfaces, respectively ($p<0.05$).

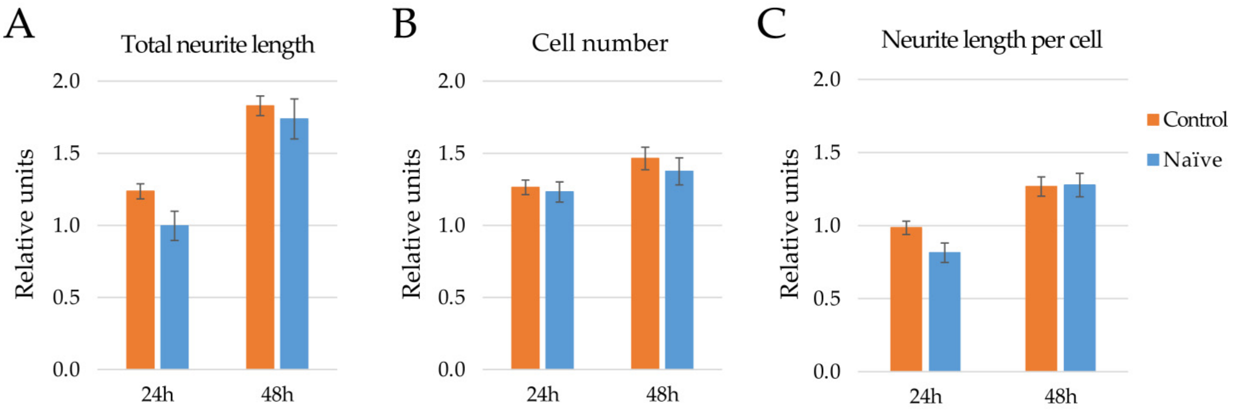

Supplementary Figure 2
Neurite generation of NPCs in the presence and absence of naïve microglia

GFP-NPCs were co-cultured with naïve BV2 cells. The total neurite lengths (A), the cell number (B) and the neurite lengths per cell (C) were determined after 24 or 48 h of co-culturing. The relative units represent the values normalized to the 0 time point of the control (NPCs with no co-culturing). Data are presented as mean ± SEM (n=8). For statistical analysis, two-way ANOVA followed by Tukey's HSD post hoc test was performed.

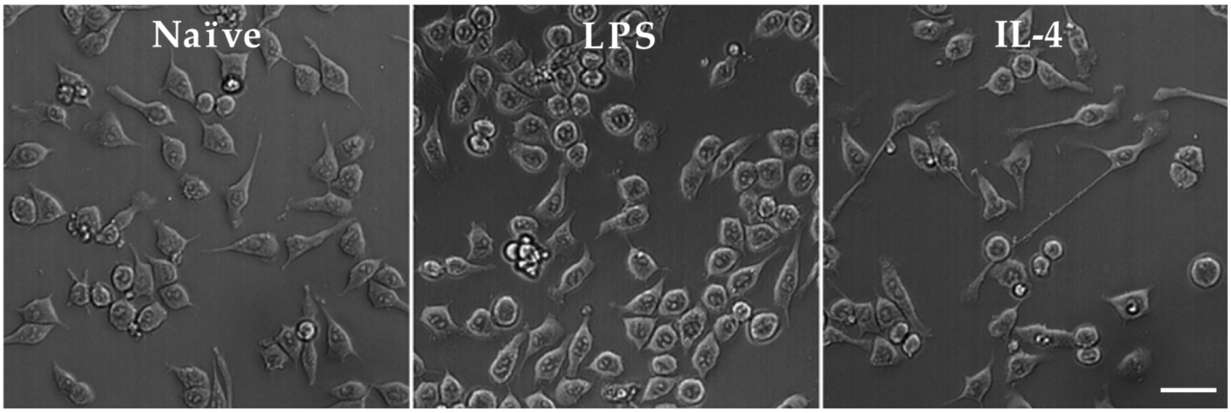

Supplementary Figure 3
Morphological changes in BV2 cells stimulated with pro- and anti-inflammatory agents

Representative bright field microscopy images of BV2 cells treated with LPS or IL-4 for 24h demonstrate marked change shape as compared to untreated microglial cells. Scale bars: 50 µm.

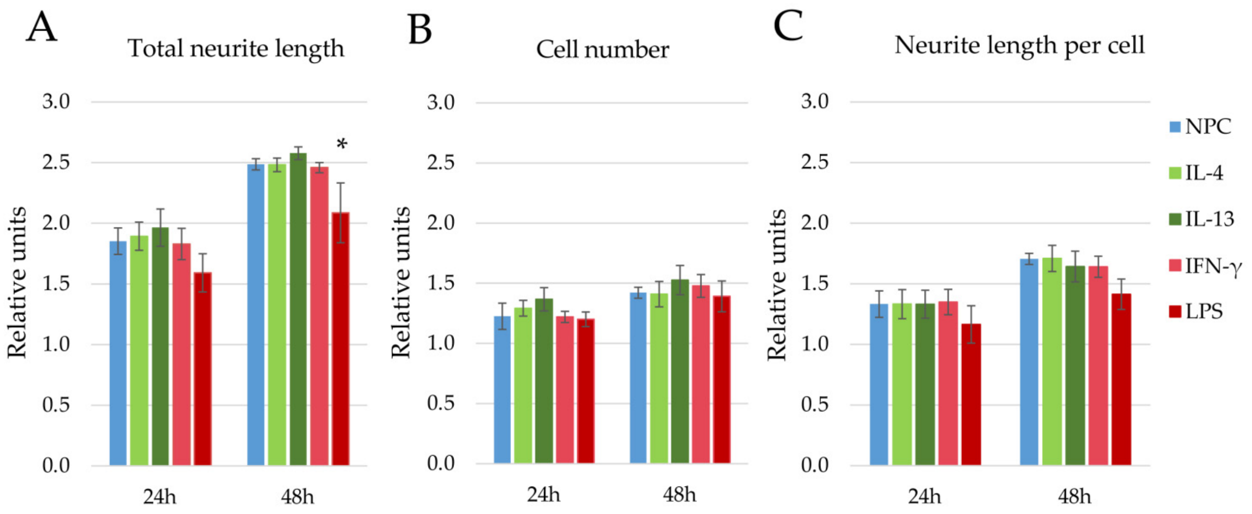

Supplementary Figure 4
Effect of pro- and anti-inflammatory agents on the proliferation and neurite generation of NPCs

GFP-NPCs were directly subjected to the previously used pro- (IFNγ, LPS) or anti-inflammatory (IL-4, IL-13) agents. The total neurite lengths (A), the cell number (B), and the neurite lengths per cell (C) were determined after 24 or 48 h. The values were normalized to time 0 points of untreated NPCs (relative units). Data are presented as mean ± SEM (n=3). For statistical analysis, two-way ANOVA followed by Tukey's HSD post hoc test was performed. Asterisk indicate the significant difference as compared to untreated NPCs ($p<0.05$).

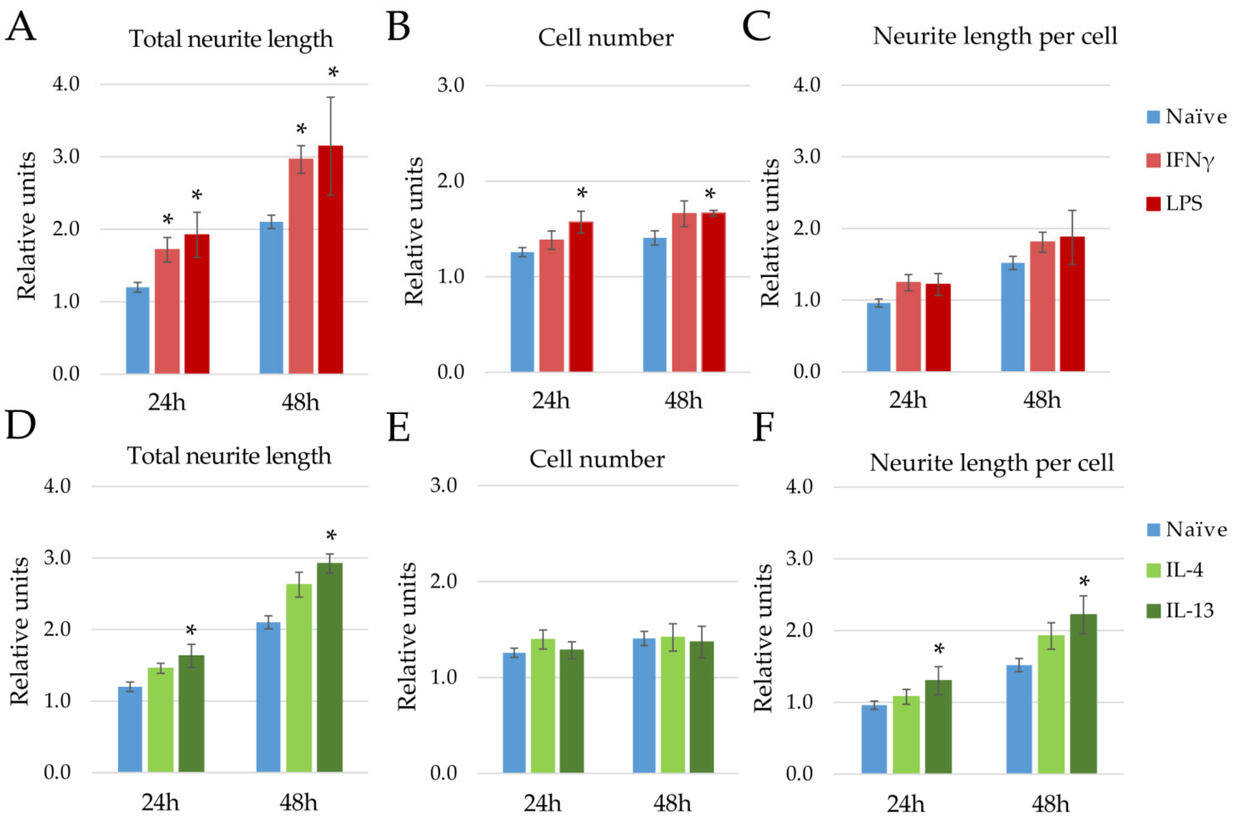

Supplementary Figure 5
Modulatory effect of microglia stimulation on microglia-NPC interaction

GFP-NPCs were co-cultured with BV2 cells previously stimulated with proinflammatory agents (IFNγ or LPS) (A-C), or anti-inflammatory cytokines (IL-4 or IL-13) (D-F). As controls, GFP-NPCs subjected to the naïve BV2 cells were used. The total neurite lengths, the cell numbers, and the neurite lengths per cell were determined at the indicated time points. Data are presented as mean ± SEM (n=4-8). For statistical analysis, two-way ANOVA followed by Tukey's HSD post hoc test was performed. Asterisks indicate significant differences as compared to co-cultures with naïve BV2 cells ($p<0.05$).

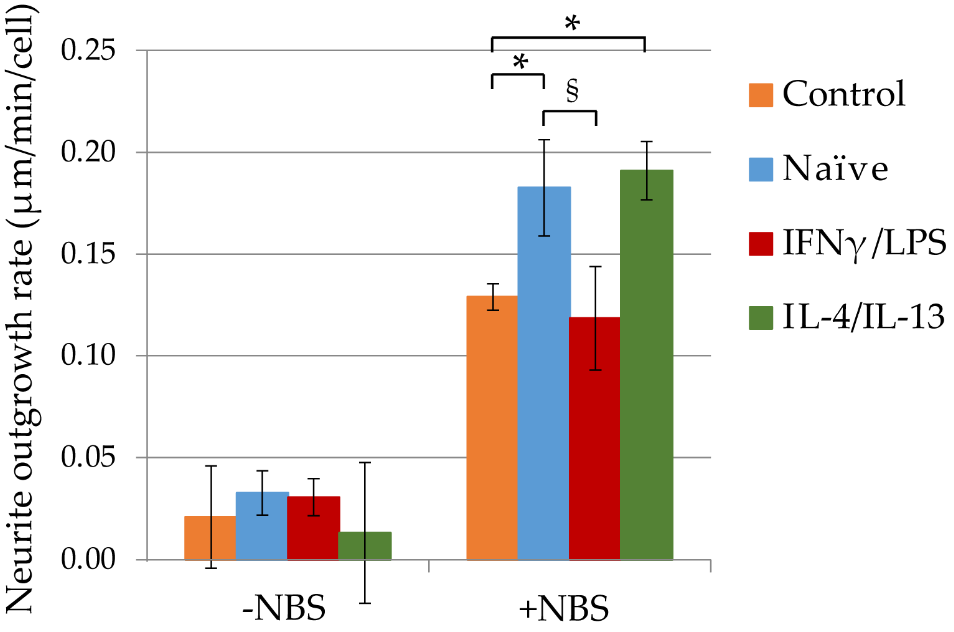

Supplementary Figure 6
Modulatory effect of microglia on para-nitroblebbistatin-induced neurite outgrowth in NPCs

Unstimulated (naïve), pro- (IFNγ/LPS) and anti-inflammatory (IL-4/IL-13) stimulated BV2 cells were co-cultured with GFP-expressing NPCs (control) for 48 h. GFP-NPCs were treated with or without 10 µM NBS and imaged for 4 h in 15-min intervals using HCS. Data are presented as mean ± SEM (n=4). For statistical analysis, two-way ANOVA followed by Tukey's HSD post hoc test was performed. The asterisks indicate significant differences as compared to control (NPCs with no co-culturing), whereas the section sign denote significant difference as compared to NPCs co-cultured with naïve microglia ($p<0.05$).

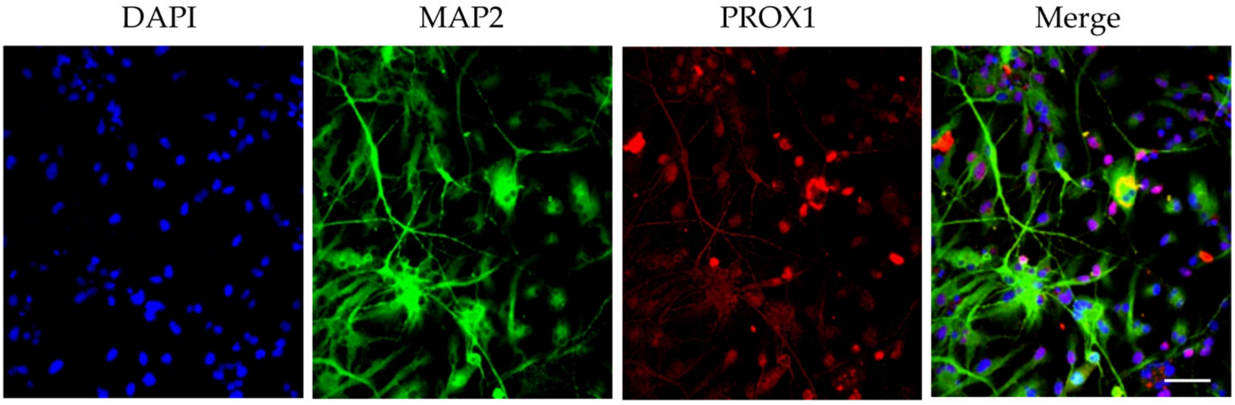

Supplementary Figure 7
PROX-1 expression in neuronal cells differentiated from human NPCs

NPCs were differentiated to the hippocampal dentate gyrus granule cells for 4 weeks. Confocal microscopy images depict the obtained neuronal cultures immunostained for MAP2 (green) and PROX1 (red), the latter of which is a hallmark of hippocampal DG granule neurons. The sample was also counterstained with DAPI (blue). The majority of cells was positive for PROX1. Scale bar: 50 μm.

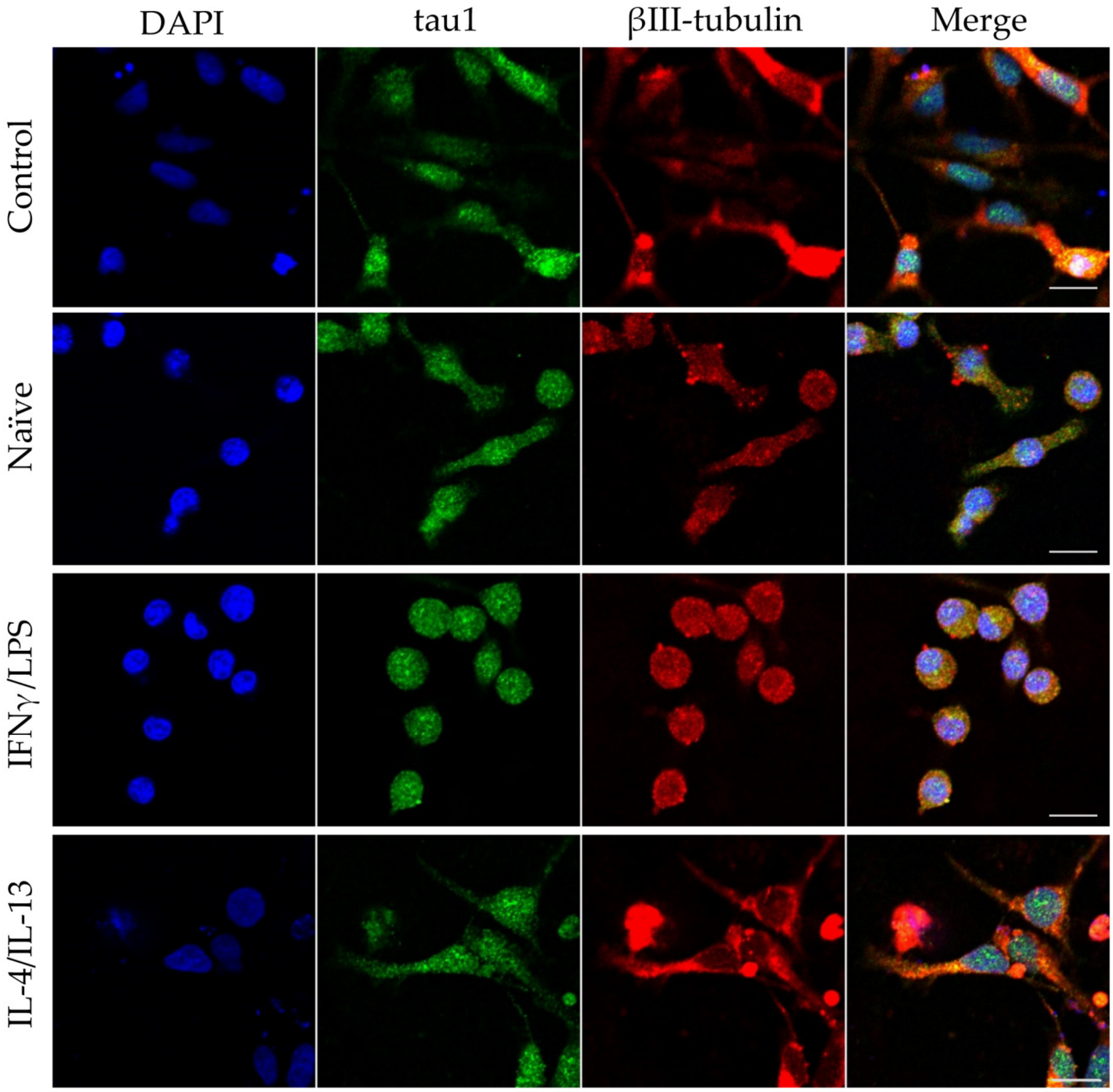

Supplementary Figure 8
Impact of naïve and stimulated microglia on the axon development in differentiating neurons at the initial phase of differentiation

Confocal images of differentiating neuronal cells exposed to naïve and stimulated BV2 cells for 4 days (from day 2 to day 6). The cultures were immunostained for the axonal marker tau1 (green), as well as for the neuronal marker βIII-tubulin (red), and counterstained with DAPI (blue) on day 6. Scale bars: 25 μm.

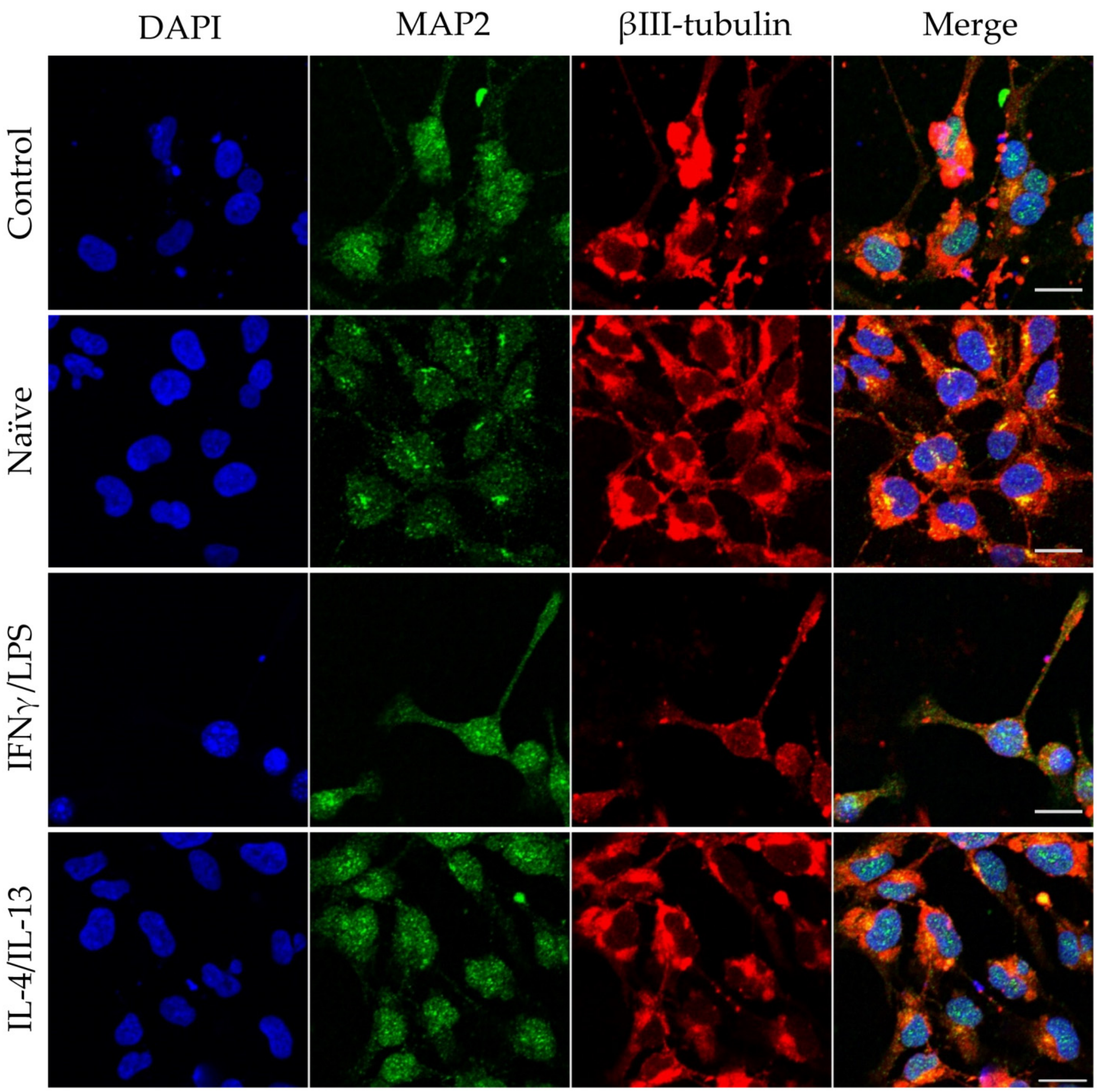

Supplementary Figure 9
Effect of naïve and stimulated microglia on the dendrite development in differentiating neurons at the initial phase of differentiation

Confocal images of differentiating neuronal cells exposed to naïve and stimulated BV2 cells for 4 days (from day 2 to day 6). The cultures were immunostained for the dendritic marker MAP2 (green), as well as for the neuronal marker βIII-tubulin (red), and counterstained with DAPI (blue) on day 6. Scale bars: 25 µm.

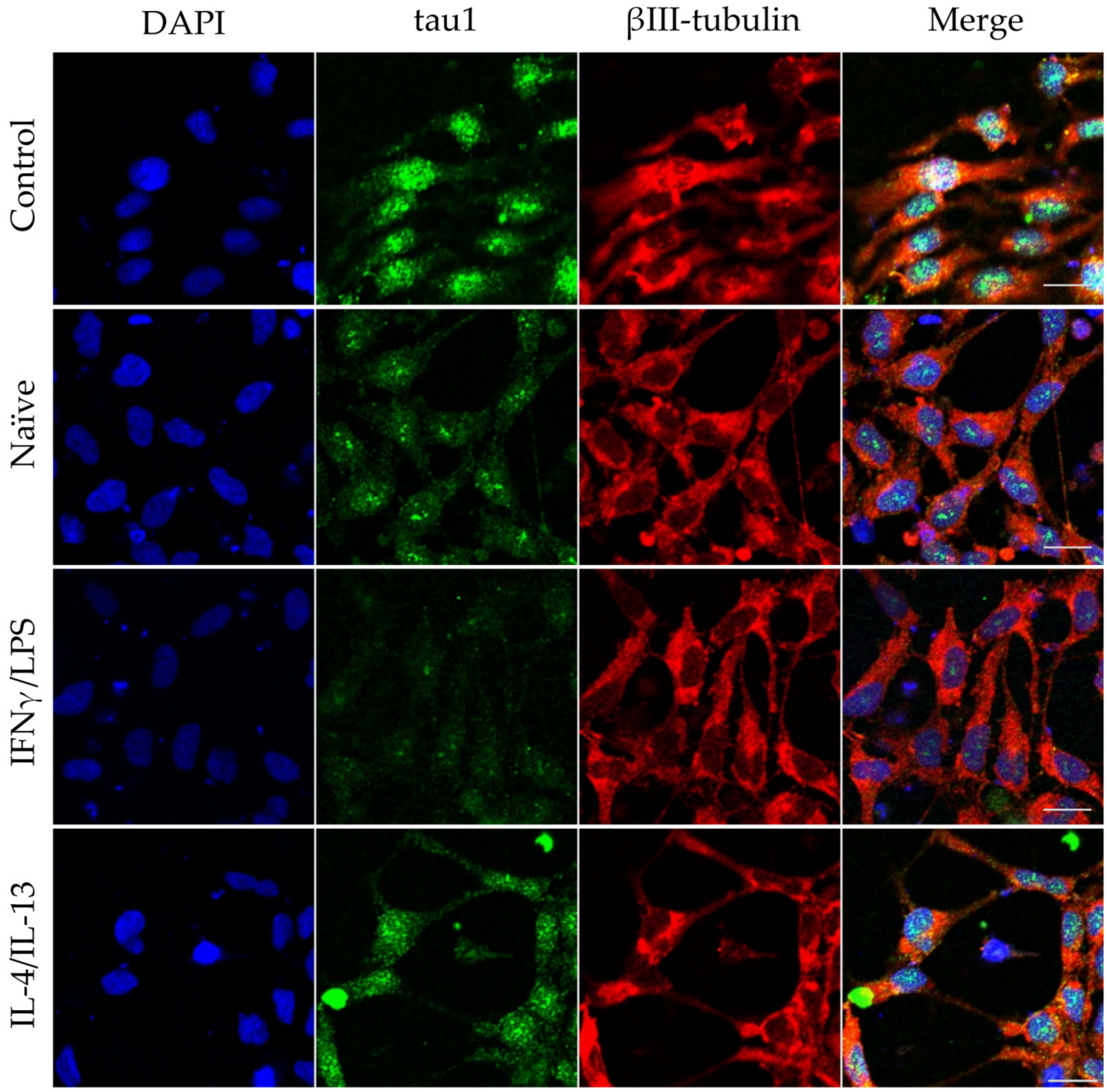

Supplementary Figure 10
Impact of naïve and stimulated microglia on the axon development in differentiating neurons at intermediate phase of differentiation

Confocal images of differentiating neuronal cells exposed to naïve and stimulated BV2 cells for 4 days (from day 8 to day 12). The cultures were immunostained for the axonal marker tau1 (green), as well as for the neuronal marker βIII-tubulin (red), and counterstained with DAPI (blue) on day 12. Scale bars: 25 µm.

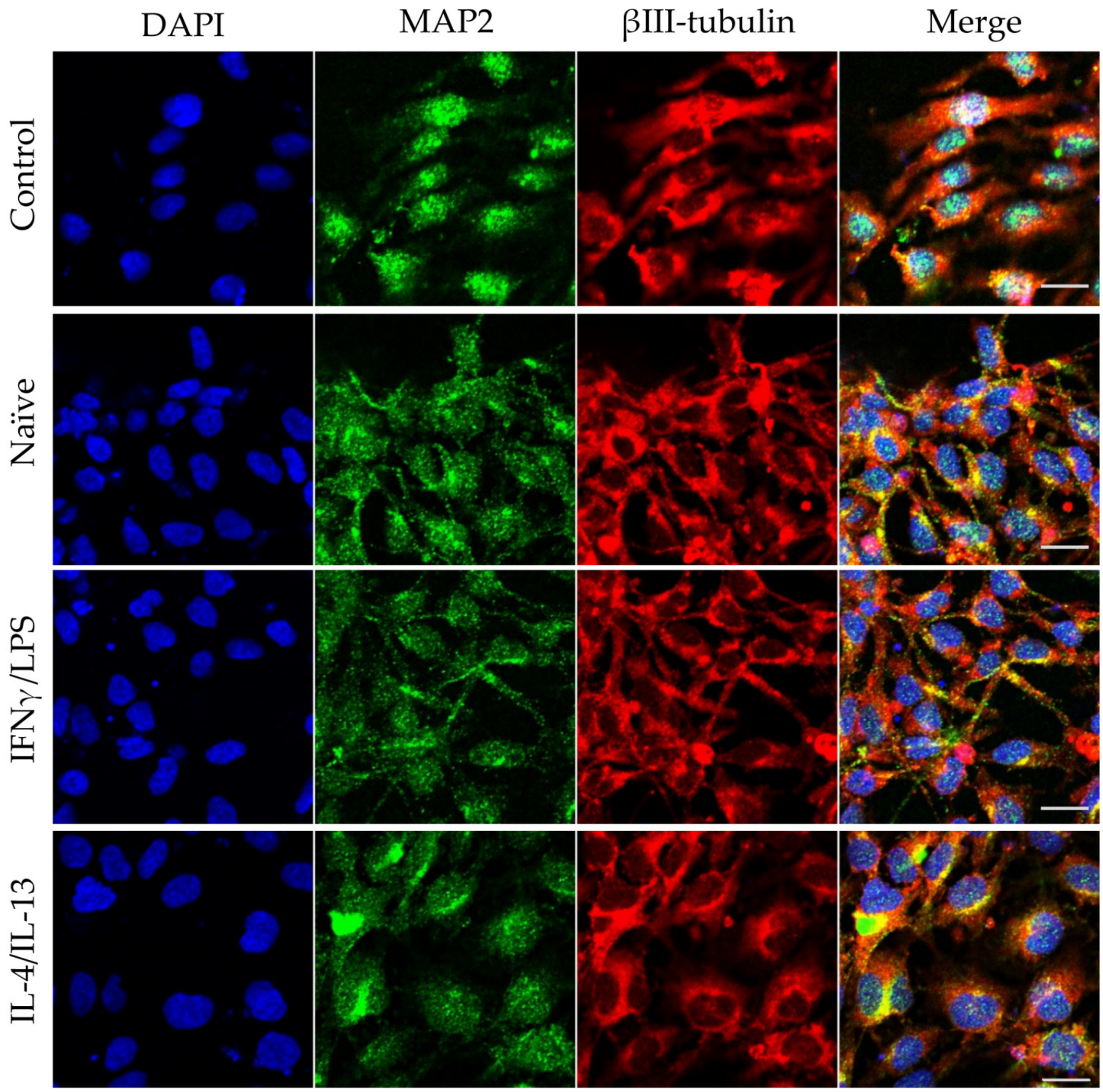

Supplementary Figure 11
Effect of naïve and stimulated microglia on the dendrite development in differentiating neurons at intermediate phase of differentiation

Confocal images of differentiating neuronal cells exposed to naïve and stimulated BV2 cells for 4 days (from day 8 to day 12). The cultures were immunostained for the dendritic marker MAP2 (green), as well as for the neuronal marker βIII-tubulin (red), and counterstained with DAPI (blue) on day 12. Scale bars: 25 µm.
